# Expression and localization of HPG axis-related genes in Carassius auratus with different ploidy

**DOI:** 10.3389/fendo.2024.1336679

**Published:** 2024-02-12

**Authors:** Xiaowei Xu, Li Yang, Xinyi Deng, Qingwen Xiao, Xu Huang, Chongqing Wang, Yue Zhou, Xiang Luo, Yuxin Zhang, Xidan Xu, Qinbo Qin, Shaojun Liu

**Affiliations:** ^1^ State Key Laboratory of Developmental Biology of Freshwater Fish, Engineering Research Center of Polyploid Fish Reproduction and Breeding of the State Education Ministry, College of Life Sciences, Hunan Normal University, Changsha, Hunan, China; ^2^ Nansha-South China Agricultural University Fishery Research Institute, Guangzhou, China; ^3^ Hunan Yuelu Mountain Science and Technology Co., Ltd., for Aquatic Breeding, Changsha, Hunan, China

**Keywords:** fertile triploid *C. auratus*, triploidization, HPG axis, methylation, immunofluorescence

## Abstract

**Introduction:**

In the Dongting water system, the *Carassius auratus* (Crucian carp) complex is characterized by the coexistence of diploid forms (2n=100, 2nCC) and polyploidy forms. The diploid (2nCC) and triploid C.auratus (3n=150, 3nCC) had the same fertility levels, reaching sexual maturity at one year.

**Methods:**

The nucleotide sequence, gene expression, methylation, and immunofluorescence of the gonadotropin releasing hormone 2(*Gnrh2*), Gonadotropin hormone beta(*Gthβ*), and Gonadotropin-releasing hormone receptor(*Gthr*) genes pivotal genes of the hypothalamic-pituitary-gonadal (HPG) axis were analyzed.

**Results:**

The analysis results indicated that *Gnrh2*, follicle-stimulating hormone receptor(*Fshr*), and Lethal hybrid rescue(*Lhr*) genes increased the copy number and distinct structural differentiation in 3nCC compared to that in 2nCC. The transcript levels of HPG axis genes in 3nCC were higher than 2nCC (P<0.05), which could promote the production and secretion of sex steroid hormones conducive to the gonadal development of 3nCC. Meanwhile, the DNA methylation levels in the promoter regions of the HPG axis genes were lower in 3nCC than in 2nCC. These results suggested that methylation of the promoter region had a potential regulatory effect on gene expression after triploidization. Immunofluorescence showed that the localization of the *Fshβ, Lhβ*, and *Fshr* genes between 3nCC and 2nCC remained unchanged, ensuring the normal expression of these genes at the corresponding sites after triploidization.

**Discussion:**

Relevant research results provide cell and molecular biology evidence for normal reproductive activities such as gonad development and gamete maturation in triploid *C. auratus*, and contribute to further understanding of the genetic basis for fertility restoration in triploid *C. auratus*.

## 1 Introduction

Crucian carp (*Carassius auratus*) belongs to the class of Teleosts, Cyprinidae, and Crucian carp (1). *C. auratus* is widely distributed worldwide due to its strong environmental adaptability and fecundity. *C. auratus* found in China can be divided into *C. auratus* and black *C. auratus* species. However, it is often stated that *C. auratus* belongs to the *C. auratus* species (2). Previous studies reported that *C. auratus* in Chinese waters were mainly diploid. However, further studies have found the coexistence of diploid, triploid, and tetraploid *C. auratus* in natural waters.

Triploid fish generally exhibit abnormal chromosomal behavior during meiosis, resulting in delayed or abnormal development of fish gonads that reduce triploid sex and infertility. Xiao et al. found that diploid, triploid, and tetraploid *C. auratus* exist in the waters of Dongting Lake, and *C. auratus* of different ploidy can stably coexist in the natural environment for a long time (2). Researchers have also found 3nCC in Dongting Lake, China. By observing the gonad tissue section of the triploid *C. auratus* (reproduction season), it was found that its gonad structure developed as expected, explaining the reproductive characteristics of triploid fertility at the cellular level.

The reproductive activity of fish is adjusted by the hypothalamis-pituitary-gonadal(HPG) (3), as well as the nervous and endocrine systems. Gonadotropin-releasing hormone (*Gnrh*), gonadotropin hormones (*Gths*), and gonadotropin receptor (*Gthr*) are key signaling molecules in the HPG axis (4). Three types of *Gnrh* in teleost (*Gnrh*1, *Gnrh2*, and *Gnrh*3) are classified according to their distribution and function (5, 6).

In some species, only two types may exist, with either *Gnrh*1 or *Gnrh*3 types missing and the function of the missing gene being complemented by the retained gene. The *Gnrh2* gene has been found in all fish studied to date, and analysis of its nucleotide sequence found that the gene was highly conserved (7, 8). In teleost, *Gnrh2* stimulates gonadotropin release, affecting animal reproductive behavior and feeding (9–11). The expression levels of *Gnrh2* were significantly altered in sterile triploid versus fertile diploid and tetraploid fish at different stages of gonad development, indicating that *Gnrh2* gene expression levels differed significantly in fish (12). The *Gnrh2* gene is an important signaling molecule on the HPG axis, and its expression level is closely related to fertility in fish.

Gonadotropin is another important signaling molecule on the HPG axis (13), and its function ensures normal gonadal development in vertebrates (14). The glycoprotein hormone *Gthβ* is produced and released by the pituitary gland and can act on the gonad to promote the production of sex steroid hormones that regulate gonad development. Studies have found that the mammalian pituitary gland contains two gonadotropins, follicle-stimulating hormone (*Fshβ*) and luteinizing hormone (*Lhβ*) (15). The *Gthβ* produced in the pituitary gland of teleost can regulate gonad development in fish. However, it has been observed that gonadotropins do not act directly on gonads. They first combine with *Gthr.* to regulate the formation and secretion of sex steroid hormones that stimulate gonad development (16). Studies have reported that *Gthr.* belongs to the G protein-coupled receptor family (17) and can be follicle-stimulating hormone receptor (*FSHR*) and luteinizing hormone receptor (*Lhr*), which share similar amino acid structures, including an N-terminal extracellular domain, a transmembrane domain, and a short C-terminal intracellular domain (18, 19). When the corresponding upstream hormone activates *Gthr.*, its transmembrane conformation domain changes (20), triggering downstream reactions. Due to abnormal chromosomal behavior during meiosis, triploids often cannot produce mature gametes with reproductive vigor, resulting in stunted or structural abnormalities in their gonads, reducing fertility or causing infertility. In this study, the coding sequence, promoter methylation level, gene expression level of genes *Gnrh2, Gthβ* (*Fshβ*, *Lhβ*), *Gthr* (*Fshr*, *Lhr*) of 2nCC, 3nCC, *Fshβ*, *Lhβ*, and *Fshr* were compared with gene expression and localization in pituitary and gonadal tissues to explore the effect of polyploidy. The effects of fertility-related genes in raw *C. auratus* provide a theoretical understanding of fertility restoration in triploid fish.

## 2 Materials and methods

### 2.1 Modification and refinement

Fish treatments were carried out according to the recommendations in the Guidelines for the Care and Use of Laboratory Animals of the National Advisory Committee for Laboratory Animal Research in China and approved by the Animal Care Committee of Hunan Normal University(Permit Number: 4237). Individual 2nCC and 3nCC were randomly selected. All samples were cultured in open pools (0.067 ha) with suitable pH (7.0-8.5), water temperature (22-24°C), dissolved oxygen content (5.0-8.0 mg/L) and adequate forage at the State Key Laboratory of Developmental Biology of Freshwater Fish, Hunan Normal University, China. All fish were anesthetized with 100 mg/L MS-222 (Sigma-Aldrich, St Louis, MO, USA) before blood collection. The study was conducted in accordance with the local legislation and institutional requirements.

### 2.2 Rearing conditions

All the *C. auratus* used in this study were from the Dongting Lake water system. After detection by flow cytometry, 2nCC and 3nCC were raised separately for subsequent collection. Two different ploidy *C. auratus* with good growth conditions during the breeding season (April) were selected for sampling. Before dissection, the experimental fish were anesthetized with 100 mg/L MS-222 (Sigma-Aldrich St. Louis MO USA), and the brains of diploid and triploid *Carassius auratus* were removed under sterile conditions. Subsequently, the pituitary, gonad, and other tissues were rapidly sectioned for RNA and DNA extraction. The RNA and DNA were then cut into small pieces, placed in EP tubes (Axygen), frozen with liquid nitrogen, and transferred to a -80°C refrigerator for histology. Sectional experimental materials were fixed with Bouin’s solution. The samples used in the experiments contained three and more biological replicates.

### 2.3 RNA isolation and cDNA synthesis

Total RNA was extracted from the brain, pituitary, and gonad of 2nCC and 3nCC in April (breeding season) under sterile and non-enzyme-contaminated experimental conditions using Total RNA Kit II from Omega. The OD value, concentration, and integrity of the extracted RNA were detected, and standard RNA was stored at -80°C. Total brain, pituitary, and gonad RNA extracted from 2nCC and 3nCC individuals were used as templates, and the PrimeScript™ RT Reagent Kit with gDNA Eraser (perfect kit for Real-Time PCR, TaKaRa RR047A) was used to generate cDNA using the reverse transcription. The cDNA obtained was stored in a -20°C refrigerator. The samples used in the experiments contained three and more biological replicates.

### 2.4 Sequence cloning of coding regions of *Gnrh2, Gthβ* and *Gthr* gene of 2nCC and 3nCC

*C. auratus* is closely related to *C. auratus red var.* We used previous research as a reference for cloning the coding regions of HPG axis-related genes in *C. auratus red var.* and autotetraploid *C. auratus* (21). The cDNA obtained by reverse transcription from the brain, pituitary, and gonad tissues of 2nCC and 3nCC was used as a starting template for cloning the coding region of the corresponding gene. PCR amplification was initiated using the primers prepared in **Table 1**, and each gene was amplified. Coding region sequences of *Gnrh2*, *Fshβ*, *Lhβ*, *Fshr*, and *Lhr* genes from two types of fish were obtained. The total reaction system was combined int 20 μL, 10 μL Premix Taq (LA Taq™ Version 2.0 plus dye) (TaKaRa Company, RR903), 1 μL of forward and reverse primers, 7 μL of sterilized double-distilled water, and 1 μL of cDNA template. The samples used in the experiments contained three and more biological replicates.

**Table 1:** Primer sequences of gene coding regions.

Primer name	Primer sequences	Annealing temperature	Extended time
*Gnrh2*-F	5’-AGTTATGGTGCACATCTGCAG-3’	55°C	30 s
*Gnrh2*-R	5’-TCACTTTCTCTTTCGGAAATCC-3’		
*Lhβ*-F	5’- GAGCAATGGGGACACCTGT-3’	56°C	30 s
*Lhβ*-R	5’- GGGGCTAGTATACAAGGAAAT-3’		
*Fshβ*-F	5’- CAGATGAGGATGCGCTTCG-3’	56.5°C	30 s
*Fshβ*-R	5’- TCTAATGTGCATTGCAGCCG-3’		
*Fshr*-F	5’- ATGACAAAGAGGATGGTCTTGT-3’	56.5°C	2 min 30s
*Fshr*-R	5’- GTCAGTACACTTGGGTGATGTG-3’		
*Lhr*-F	5’- AAGACAATACGGGACAATGC-3’	53.5°C	2 min 30s
*Lhr*-R	5’- ATGAGTCTGAATGTCCGTTTC-3’		

The amplification reaction procedures for each gene were generally similar. PCR products obtained were detected with electrophoresis. When fragments of the desired size were found, they were recovered using gel. For detailed experimental procedures, refer to the attached instruction manual for the DNA gel recovery kit (Shanghai Sangong, B518131-0100). The products were ligated and transformed, and a single colony was selected from the ampicillin-resistant petri dish (30 per petri dish) for PCR. The total reaction system was 10 μL, and 1 μL of the bacterial solution was taken as the starting point Template, 5 μL of 2× Rapid Taq Master Mix (Vazyme, P222-AA), 3 μL of sterilized double-distilled water, 0.5 μL of forward and reverse primers. The primers are specific for the corresponding genes in **Table 1**. The product was detected, and the bacterial liquid similar to the target fragment was screened to complete the detection. Twenty samples for each gene were sent for detection to reduce the error caused by the small number of sequences.

### 2.5 Analysis of *Gnrh2*, *Gthβ* and *Gthr* gene expression and methylation levels in promoter regions of 2nCC and 3nCC

Quantitative reverse transcription PCR (RT-qPCR) was used to detect mRNA transcript levels of HPG axis-related genes in 2nCC and 3nCC. Specific RT-qPCR primers were designed to code region sequences of HPG axis-related genes in 2nCC and 3nCC. The primer sequences are shown in **Table 2**. *β-actin* is the internal reference gene. The cDNA of each tissue obtained in 2.2 was diluted with sterile water at a ratio of 1:5 and used as the starting template for RT-qPCR. The total reaction system was 10 μL, of which 2x Power SYBR Green PCR Master Mix (ABI) 5 μL sterilized water 3 μL, forward and reverse primers 0.5 μL each, and diluted cDNA 1 μL. Fluorescence assay was performed using the Prism7500 Sequence Detection System (ABI), and biological replicates were performed to ensure reliable experimental results.

**Table 2:** Primer sequences of *Gnrh2, Gthβ* and *Gthr* genes by RT-qPCR.

Primer name	Primer sequences
Q-*Gnrh2*-F	5’-AAGTGCCCAGTTTGCCAG-3’
Q-*Gnrh2*-R	5’-TCGGAAATCCCGTGTGAG-3’
Q-*Lhβ*-F	5’- GCTTGCCAGACTGTCCTC-3’
Q-*Lhβ*-R	5’- GTCAGATGTGTCCATAGTGC-3’
Q-*Fshβ*-F	5’- CTGTCGGCTCACCAATATCTCC-3’
Q-*Fshβ*-R	5’- CGTCCATTCTCTGAAGTTAC-3’
Q-*Fshr*-F	5’- ATTCCTGCTCGAACCCGTTT-3’
Q-*Fshr*-R	5’- CTCTGTGCGGTAAATGTGCG-3’
Q-*Lhr*-F	5’-TCAACGTTCTGGCCATCGTC-3’
Q-*Lhr*-R	5’-CGTGTCATGAGATCCACGGT-3’
*β-actin*-F	5’-GCCCTGCCCCATGCCATCCT-3’
*β-actin*-R	5’-AGTGCCCATCTCCTGCTCGA-3’

The DNA of each prepared tissue was treated with an EZ DNA Methylation-Gold kit (D5005, ZYMO RESEARCH, USA). The CT Conversion Reagent solution was prepared to collect the total DNA (400 ng) from each tissue. Subsequently, 20 μL of sterilized water was added to a 200 μL PCR tube, and then 130 μL of the prepared CT Conversion Reagent solution was added to the PCR tube and mixed well. The above-mentioned mixed PCR samples were put into the PCR machine, and the set program was 98°C for 10 min and 64°C for 150 min. After PCR, the product was temporarily stored at 4°C for 20 h. Subsequently, 600 μL of M-Binding Buffer was first added to the adsorption column, followed by the processed PCR samples, and they were mixed by inversion after closing the tube cap. The samples were then centrifuged, and the filtrate was poured off. Then 200 μL M-wash Buffer was drawn and added to the adsorption column, centrifuged, and the filtrate was poured off. Lastly, 200 μL M-Desulphonation Buffer was drawn and put in the adsorption column, centrifuged, and the filtrate was poured off. Subsequently, M-Desulphonation Buffer was drawn and put into M-Elution Buffer heated in a water bath at 56°C. Then 16 μL of the solution was pipetted into the adsorption column, incubated at room temperature for 15 min, centrifuged, and the obtained DNA was stored at -20°C. The samples used in the experiments contained three and more biological replicates.

Based on the mRNA sequences of *C. auratus red var. Gnrh2*, *Fshβ*, *Lhβ*, *Fshr*, and *Lhr* genes in the GenBank database, the complete DNA sequences of the above genes were searched from the *C. auratus red var.* genome. Then, the mRNA and DNA sequences of these genes were compared. When the first exon was found, the upstream 2000 bp sequence was used as the promoter region of the gene, and the sequence of the selected region was entered into the Meth Primer online website and predicted CpG-rich regions in the promoter regions of *Gnrh2*, *Fshβ*, *Lhβ*, *Fshr*, and *Lhr* genes. The CpG-enriched region of each gene (if a single gene has multiple CpG-enriched regions, randomly select a region from them) was selected as the original target sequence to design primers. The primer sequences are shown in **Table 3**. They are used to amplify CpG-enriched promoters of the corresponding 2nCC and 3nCC genes. The bisulfite PCR (BS-PCR) primers were designed with Primer Premier 5.0 software using the CpG-enriched region of the HPG axis-related gene promoter cloned from *C. auratus* with a different ploidy than the original target sequence. BS-PCR was performed using DNA from 2.2 as a template and the specific primers in **Table 4**. The total reaction system was 10 μL of LA DNA polymerase 5 μL, 0.5 μL forward and reverse primers, 3.5 μL sterilized water each, and 0.5 μL of template DNA. The resulting cloned products were subjected to electrophoresis analysis, and strips containing the expected fragment length were subjected to gel recovery followed by ligation, transformation, and sequencing.

**Table 3:** Primer sequences of *Gnrh2, Gthβ* and *Gthr* genes promoter region.

Primer name	Primer sequences
*Gnrh2*-F	5’-GAGTCAGTCTTACTCTGT-3
*Gnrh2*-R	5’-TATATATTTTTCAACCAT-3
*Fshβ*-F	5’-TCAGACAGAAGCATTTTG-3
*Fshβ*-R	5’-GTCTGGCTCTATGGCTTT-3
*Lhβ*-F	5’-TACAAACACTAATGAACT-3
*Lhβ*-R	5’-AGGTGTCCCCATTGCTCA-3
*Fshr*-F	5’-TAACCACCCTAAGAGTCC-3
*Fshr*-R	5’-CAGACACTGACACCAAAC-3
*Lhr*-F	5’-ATGACTGATTCTTTGTTG-3
*Lhr*-R	5’-GGGAGAAGACCTCACAAA-3

**Table 4:** Primer sequences of *Gnrh2, Gthβ* and *Gthr* genes by BS-PCR.

Primer name	Primer sequences
M-*Gnrh2*-F	5’-TTGCGATGAGTTAGTTTTATTTTG-3’
M-*Gnrh2*-R	5’-TATTTTTCAACCATAACAACTCCA-3’
M-*Fshβ*-F	5’-TTGATGGGAGTGAAAAGATAGA-3’
M-*Fshβ*-R	5’-TAACTTTTCATCTCCAACTCAA-3’
M-*Lhβ*-F	5’-AAAGTGTTTTAGTGTTTATTGT-3’
M-*Lhβ*-R	5’-CCCATTACTCAACAAACTATTA-3’
M-*Fshr*-F	5’-TGAAATGAGAAGAGATTGAGAAAG-3’
M-*Fshr*-R	5’-CGTATTCAAACACTAACACCAAAC-3’
M-*Lhr*-F	5’-ATGATTGATTTTTTGTTGTGTA-3’
M-*Lhr*-R	5’-TTCACGAAATCAAATCTAAAAA-3’

### 2.6 Expression and localization of *Fshβ*, *Lhβ* and *Fshr* in 2nCC and 3nCC

The pituitary, ovary, and testis tissues of 2nCC and 3nCC in the breeding season (April) were removed and soaked in Bouin’s solution for 48 h. After 48 h, small tissues were separated (pituitary tissues do not need to be separated), and the excess fixative was blotted on the surface with filter paper. The separated tissues were placed in gradient alcohol for paraffin embedding. The dehydration time and alcohol gradient are 70% alcohol for 1 h (2-3 times, observable 80% alcohol, overnight; 90%, 95%, 100% alcohol, 30 min each in turn). After alcohol gradient dehydration, the materials were transferred to xylene solution and allowed to stand for 5-10 min. Subsequently, the paraffin was melted and put in an oven at about 60°C for 2-3 h to ensure the paraffin was in a molten liquid state. The tissues soaked in wax were moved to an embedding rack, and paraffin was allowed to cool. Subsequently, the tissue block was removed from the packaging. The wax was removed from the embedded rack and trimmed, and serial sections with a thickness of about 5-7 mm were prepared using a microtome. The cut wax slice was flattened with warm water, removed from the glass slide, and placed in a drying machine (42°C, 24-48 h). After drying, they were put in the slide box and prepared for subsequent experiments. The samples used in the experiments contained three and more biological replicates.

The prepared pituitary tissue sections were placed in xylene solution for dewaxing. After deparaffinization, they were rehydrated in gradient alcohol at 100%, 95%, 90%, 80%, and 70%, each for 5 min. The 0.5% periodic acid solution was oxidized for 5 min. After oxidation, the sections were stained with Schiff reagent (Shanghai Soleibao Biological Co., Ltd.) for 15 min and rinsed with running water for about 10 min. Subsequently, the sections were stained with 1% orange-yellow G Solution and 5% phosphotungstic acid solution for 10-15 s and rinsed with running water for 15 s. The sections were then stained with 1% methyl blue solution for 2-5 s and washed with 1% acetic acid solution for 1-5 s. Finally, the sections were dehydrated in gradient alcohol 70%, 80%, 90%, 95%, and 100% for 5 min each. The xylene was transparent for 5 min, mounted with gum, and then observed and photographed with a microscope (Olympus CX41).

The prepared ovary and testis tissue sections were put in xylene solution for dewaxing. After Deacetylation and rehydration were consistent with the above experiments; After rehydration, the tissues were stained with the hematoxylin staining solution for 25 min-1 h. The tissues were then rinsed and soaked in the glacial acetic acid solution for about 5-10 s, and color changes were observed. When the tissues turned red, they were removed from the stain and washed with running water. Subsequently, the slices were soaked in an alkaline aqueous solution for about 5-10 s, and color changes were observed. Tissues were removed from the stain once they turned blue, washed with running water, and dehydrated in 70%, 80%, and 90% graded alcohol for 5 min each. After dehydration, the sections were put in an eosin staining solution for 5-20 min. The sections were then transferred to 95% and 100% alcohol solution and allowed to stand for 5 min for dehydration. Subsequently, the sections were transferred to xylene for about 4-5 min and sealed with gum. The residual mounting medium was wiped with xylene, observed, and photographed with an Olympus CX41 microscope.

The immunofluorescence technique analyzed the localization of *Fshβ* and *Lhβ* in the pituitary tissue of crucian diploid carp. Immunofluorescence technology detects the expression and localization of proteins and polypeptides in biological cells or tissues using the principle that antigens can be specifically linked to antibodies (21). The reagents required included rabbit anti-*Lhβ* (primary antibody), rabbit anti-*Fshβ* (primary antibody), donkey anti-rabbit IgG labeled with FITC (fluorescent secondary antibody), immuno-highlighter, 20xPBS (phosphate buffered saline, purchased from BBI Bio), anti-degreasing slides and polylysine-treated anti-fluorescence quenchers, Triton-X 100 purchased from Shanghai Sangon Bio Co., Ltd., blocking buffer BSA and peroxidase blocking solution. The other reagents needed were prepared in the laboratory. The negative control was prepared using diluted PBS instead of the primary antibody. The pituitary tissue section obtained in 2.6 were washed three times with 1xPBS, 5 min/time. Subsequently, excess 1xPBS on the section was removed, and peroxidase blocking solution was added dropwise.

Sections were placed in a wet box at room temperature for 30 mins and then washed thrice with 1xPBS for 5 minutes each. The surrounding tissue material was wiped, and Triton-X 100 (0.5%) was added dropwise to soak the material and placed in the wet box at room temperature for 40 min. The tissues were then washed with 1xPBS three times, 5 min/time. After washing, microwave high-temperature treatment was used to repair the antigen of the test object. Subsequently, the test tissue was placed in citric acid antigen retrieval solution for high-temperature treatment for 15 min and then placed at room temperature for 1-2 min. After incubation, the slices were washed thrice with 1xPBS, 5 min/time. Then BSA blocking solution was added to the tissue material and stored in a humid box at room temperature for about 1-2 h. After 2 h, the slices were removed from the box, wiped, and used as an immunohighlight pen.

The tissue on the same slice was divided into different sections. The diluted primary antibody (the primary antibody was diluted with 1xPBS at a ratio of 1:150) and 1xPBS buffer (negative control) were added dropwise. The slice was placed in the wet box and stored light shield box. The slices were hybridized at 4°C overnight (12-14 h) to recover the primary antibody. The recovered antibody was incubated at 37°C for 30 min and washed thrice with 1xPBS, 5 min/time. Subsequently, diluted fluorescent two antibody (dilute the secondary antibody at a ratio of 1:150 with 1xPBS) was dropped into the antibody in a dark environment. The slices were then hybridized at room temperature for 1-2 h in a humidified dark box, washed thrice with 1xPBS, 5 min/time (washing was performed in a dark environment). Excess buffer and added anti-fluorescence quencher were removed under dark conditions. Subsequently, the slices were placed under a fluorescence microscope (Olympus BX63) to observe and photograph the tissue fluorescence site. The immunofluorescence technique was used to observe the localization of *Fshr* in the ovaries and testis of 2nCC.

### 2.7 Analysis and statistical testing

The fluorescence experiments mentioned in the manuscript were performed using a Prism7500 Sequence Detection System (ABI) and biological replicates were performed to ensure the reliability of the results. The results were viewed using the widely recommended relative standard curve method (2^-ΔΔCT^ method), and the experimental values were statistically calculated using Excel, analyzed for significance using SPSS 19.0 software, and finally plotted using Sigma Plot 12.5 software.

## 3 Results

### 3.1 Analysis of sequence cloning of coding regions of *Gnrh2, Gthβ* and *Gthr* gene

Coding region sequences from *Gnrh2*, *Fshβ*, *Lhβ*, *Fshr*, and *Lhr* genes were cloned into 2nCC and 3nCC. The *Gnrh2* gene contains two diploid copies, *Gnrh2*-2nCC-1 and *Gnrh2*-2nCC-2, and three triploid copies, *Gnrh2*-3nCC-1 and *Gnrh2*-3nCC-1 and *Gnrh2*-3nCC-3. The length of the *Gnrh2* gene encoding region in both fish is 261 bp and encodes 86 amino acids. The *Fshr* gene contains two diploid copies, *Fshr*-2nCC-1 and *Fshr*-2nCC-2, and three triploid copies, *Fshr*-3nCC-1 and *Fshr*-3nCC-1 and *Fshr*-3nCC-3. The length of coding regions of the *Fshr* gene in both fish is 2013 bp and encodes 670 amino acids. The *Lhr* gene contains two copies of the diploid called *Lhr*-2nCC-1 and *Lhr*-2nCC-2.

The length of the gene coding region is 2127 bp and encodes 708 amino acids; it contains three copies of the triploid named *Lhr*-3nCC-1, *Lhr*-3nCC-2, and *Lhr*-3nCC-3. The length of the gene coding regions were 2124 bp, 2127 bp, and 2127 bp, encoding 707, 708, and 708 amino acids, respectively. In both diploid and triploid, there is only one copy of the *Fshβ* gene, and the consensus length of the gene coding region is 393 bp, encoding 130 amino acids. There is only one copy of the *Lhβ* gene in two different ploidy fish, and the consensus length of the gene coding region is 423 bp and encodes 140 amino acids. Using BioEdit software to analyze the similarity of amino acid sequence and nucleotide sequence of HPG axis-related genes in 2nCC and 3nCC, it was found that the similarity of the *Fshβ* and *Lhβ* genes of the two fish was 100%. It was also observed that some base sites of the coding regions of *Gnrh2*, *Fshr*, and *Lhr* genes were mutated, and some bases were deleted in the *Lhr* gene coding region.

**Figure 1:**
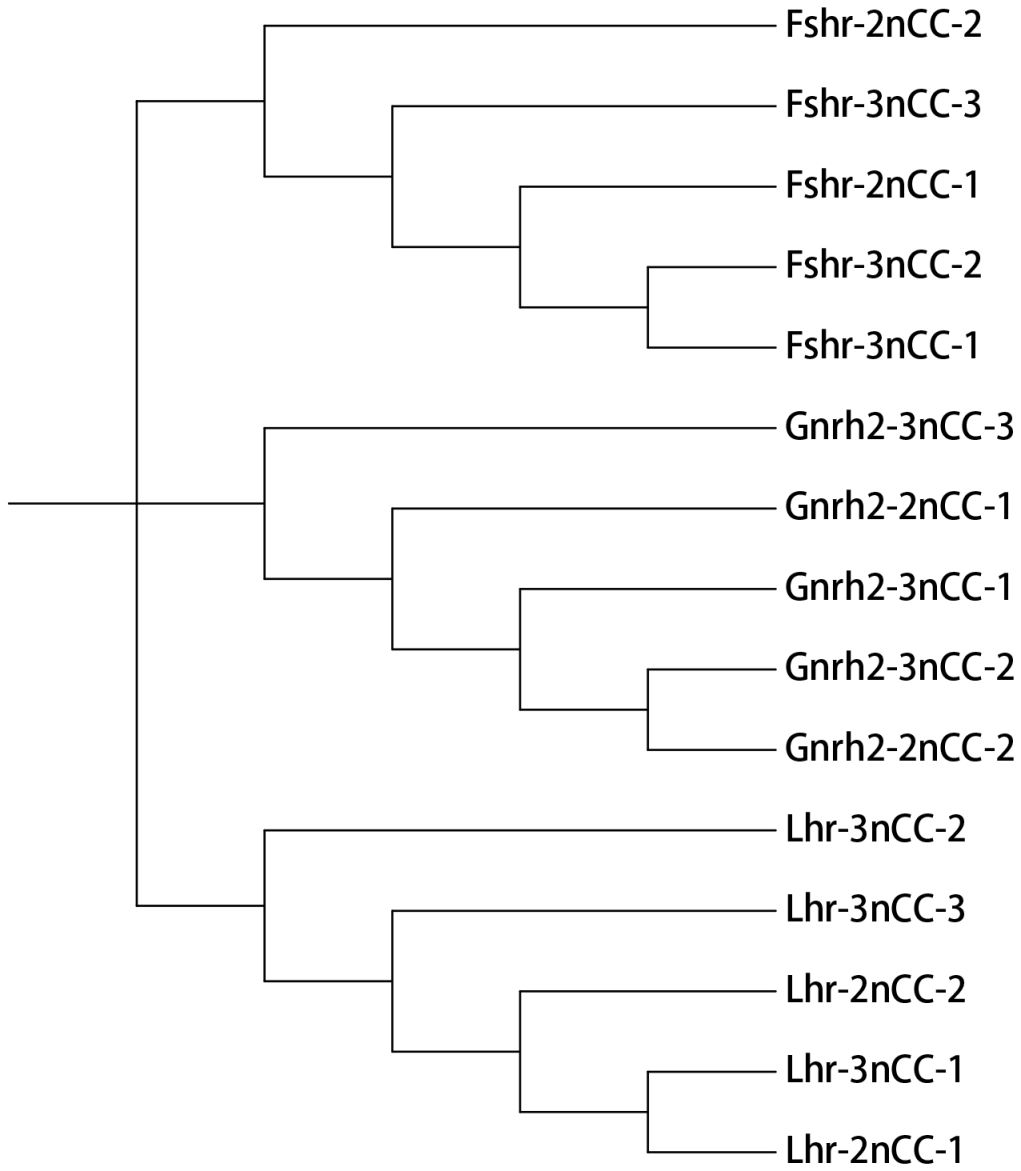
Phylogenetic tree construction using *Gnrh2*, *Fshr*, and *Lhr* genes of 2nCC and 3nCC.

**Figure 2:**
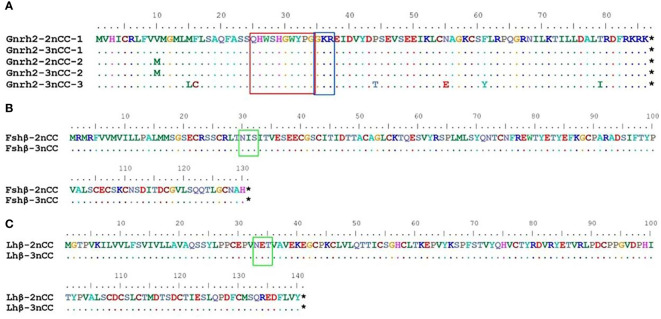
Comparison of amino acid sequences encoded by *Gnrh2, Fshβ, Lhβ* genes in 2nCC and 3nCC. **(A)** Comparison of amino acids encoded by the *Gnrh2* gene in 2ncc and 3ncc. **(B)** Comparison of amino acids encoded by the *Fshβ* gene in 2ncc and 3ncc. **(C)** Comparison of amino acids encoded by the *Lhβ* gene in 2ncc and 3ncc. Comparison of amino acids encoded by the *Gnrh2* gene in 2ncc and 3ncc The red box shows the biologically active decapeptide of *Gnrh2*; the blue box shows the proteolytic site of *Gnrh2*; the green box shows the glycosylation site of *Fshβ* and *Lhβ*. * stands for stop codon.

**Figure 3:**
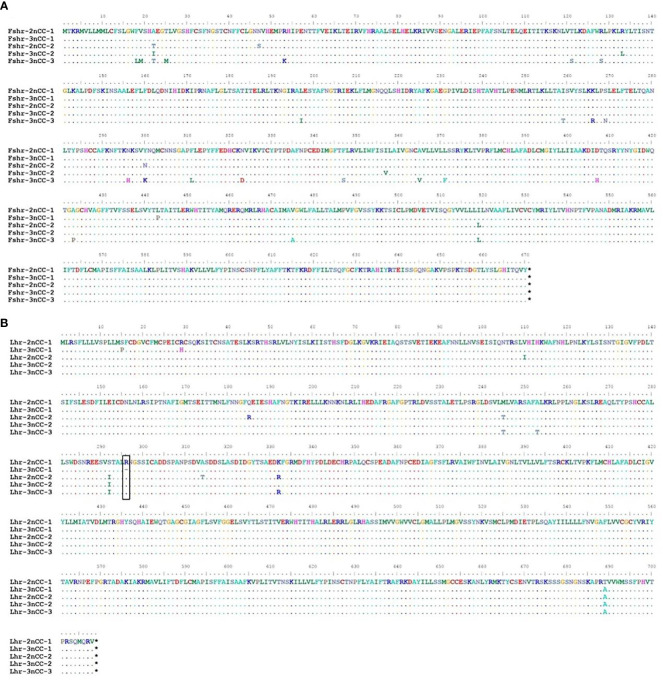
Comparison of amino acid sequences encoded by *Fshr, Lhr* genes in 2nCC and 3nCC. **(A)** Comparison of amino acids encoded by the *Fshr* gene in 2ncc and 3ncc. **(B)** Comparison of amino acids encoded by the *Lhr* gene in 2ncc and 3ncc. The black box shows the amino acid deletion site of *Lhr*. * stands for stop codon.

MEGA11 software was used to analyze the developmental relationship of *Gnrh2*, *Fshr*, and *Lhr* genes copies in 2nCC and 3nCC, and the genetic homology between the two was represented using evolutionary trees (**Figure 1**). Analysis of the *Gnrh2* gene showed that *Gnrh2*-2nCC-1 and *Gnrh2*-3nCC-1 had the same sequence, *Gnrh2*-2nCC-2 had the same sequence as *Gnrh2*-3nCC-2, and *Gnrh2*-3nCC-3 was similar to *Gnrh2*-2nCC-1. Closer analysis of the *Fshr* gene showed that the sequence similarity was greatest between *Fshr*-2nCC-1 and *Fshr*-3nCC-1 and between *Fshr*-2nCC-2 and *Fshr*-3nCC-2. The results also showed the greatest sequence similarity between *Fshr*-3nCC-3 and *Fshr*-2nCC-2. Analysis of the *Lhr* gene showed that the sequence similarity between *Lhr*-2nCC-1 and *Lhr*-3nCC-1 and between *Lhr*-2nCC-2 and *Lhr*-3nCC-2 was relatively close. In contrast, *Lhr*-3nCC-3 and *Lhr*-2nCC-2 had the closest sequence similarity. **Table 5** shows the sequence and amino acid sequence of gene coding regions related to the HPG axis. Amino acid sequence analysis (**Figures 2**, **3**) found that the *Gnrh2* amino acid sequences of 2nCC and 3nCC both contain highly conserved biologically active decapeptides and proteolytic sites; both can be found in the *Fshβ* genes of two different ploidy carp. There are 12 cysteine residue sites and a highly conserved glycosylation site, “NIS”; in their *Lhβ* genes, there are 12 cysteine residue sites and a highly conserved glycosylation site, “NET.”

**Table 5:** Comparison of amino acid sequence similarity (A%) and nucleotide sequence similarity (B%) of HPG axis-related genes in 2nCC and 3nCC.

Gene			Length		A%	Amino acid		B%
*Gnrh2*	2nCC-1			2nCC-1:2nCC-2	99.60%		2nCC-1:2nCC-2	98.80%
				2nCC-1:3nCC-1	100%		2nCC-1:3nCC-1	100%
			
				2nCC-1:3nCC-2	99.60%		2nCC-1:3nCC-2	98.80%
				2nCC-1:3nCC-3	94.20%		2nCC-1:3nCC-3	93.10%
				2nCC-2:3nCC-1	99.60%		2nCC-2:3nCC-1	98.80%
	2nCC-2		261	2nCC-2:3nCC-2	100%	86	2nCC-2:3nCC-2	100%
				2nCC-2:3nCC-3	93.80%		2nCC-2:3nCC-3	91.90%
				3nCC-1:3nCC-2	99.60%		3nCC-1:3nCC-2	98.80%
	3nCC-1			3nCC-1:3nCC-3	94.20%		3nCC-1:3nCC-3	93.10%
	3nCC-2							
	3nCC-3			3nCC-2:3nCC-3	93.80%		3nCC-2:3nCC-3	91.90%
*Fshβ*-2nCC	* *							
			393		100%	130		100%
*Fshβ*-3nCC	* *							
*Lhβ*-2nCC	* *							
			423		100%	140		100%
*Lhβ*-2nCC	* *							
*Fshr*	2nCC-1		2013	2nCC-1:2nCC-2	98.60%	670	2nCC-1:2nCC-2	99.40%
				2nCC-1:3nCC-1	99.40%		2nCC-1:3nCC-1	99.80%
				2nCC-1:3nCC-2	99.40%		2nCC-1:3nCC-2	99.50%
				2nCC-1:3nCC-3	96.00%		2nCC-1:3nCC-3	96.70%
				2nCC-2:3nCC-1	98.50%		2nCC-2:3nCC-1	99.20%
	2nCC-2			2nCC-2:3nCC-2	98.70%		2nCC-2:3nCC-2	99.10%
				2nCC-2:3nCC-3	96.30%		2nCC-2:3nCC-3	96.80%
	3nCC-1			3nCC-1:3nCC-2	99.40%		3nCC-1:3nCC-2	99.40%
				3nCC-1:3nCC-3	96.00%		3nCC-1:3nCC-3	96.50%
	3nCC-2							
				3nCC-2:3nCC-3	96.20%		3nCC-2:3nCC-3	96.40%
	3nCC-3							
*Lhr*	2nCC-1			2nCC-1:2nCC-2	99.30%		2nCC-1:2nCC-2	99.00%
				2nCC-1:3nCC-1	99.40%		2nCC-1:3nCC-1	99.20%
				2nCC-1:3nCC-2	99.60%		2nCC-1:3nCC-2	99.70%
			2127	2nCC-1:3nCC-3	99.20%	708	2nCC-1:3nCC-3	99.20%
				2nCC-2:3nCC-1	99.10%		2nCC-2:3nCC-1	98.50%
	2nCC-2			2nCC-2:3nCC-2	99.40%		2nCC-2:3nCC-2	99.20%
				2nCC-2:3nCC-3	99.20%		2nCC-2:3nCC-3	99.40%
				3nCC-1:3nCC-2	99.40%		3nCC-1:3nCC-2	99.20%
	3nCC-1		2124			707	
				3nCC-1:3nCC-3	99.10%		3nCC-1:3nCC-3	98.80%
	3nCC-2							
			2127	3nCC-2:3nCC-3	99.20%	708	3nCC-2:3nCC-3	99.50%
	3nCC-3							

### 3.2 RT-qPCR results

**Figure 4:**
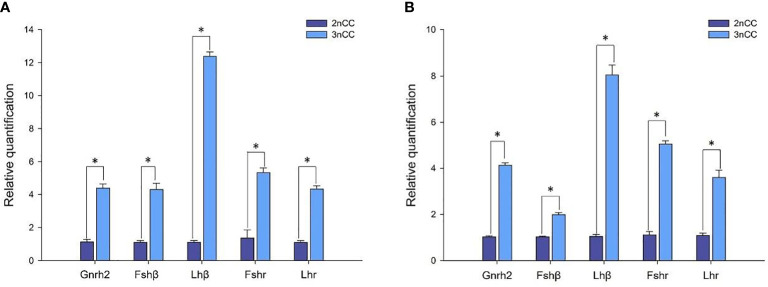
Expression levels of HPG axis-related genes in 2nCC and 3nCC. **(A)** 2nCC and 3nCC (female); **(B)** 2nCC and 3nCC (male); * indicates significant difference (*P*<0.05).

Quantitative Real-time PCR for the breeding season (April) 2nCC and 3nCC *Gnrh2, Gthβ* (*Fshβ*, *Lhβ*) and *Gthr* (*Fshr*, *Lhr*) genes were used to detect the mRNA transcription levels, and the results obtained are as shown in **Figure 4**. Epigenetic expression levels of the *Gnrh2*, *Fshβ*, *Lhβ*, *Fshr*, and *Lhr* genes in both female and male 3nCC were significantly higher than those of the corresponding genes in 2nCC during the same period. The p-value for the expression level was *P*<0.05 (**Figure 4**).

### 3.3 BS-PCR results

**Figure 5:**
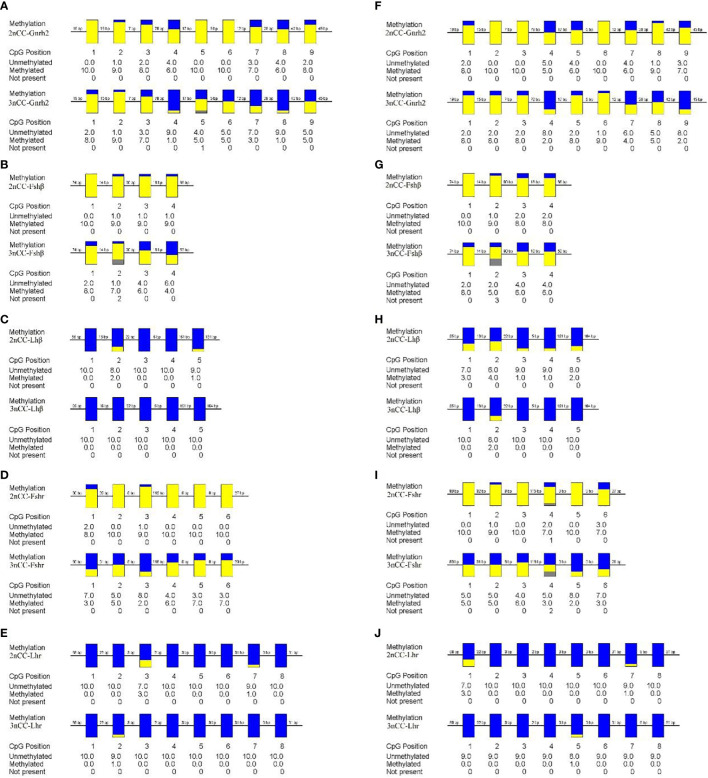
Methylation levels of HPG axis-related gene promoters in 2nCC and 3nCC. **(A–E)** 2nCC and 3nCC (female); **(F-J)** 2nCC and 3nCC (male); yellow represents methylation; blue represents unmethylated; gray represents deletion.

To study the relationship between the expression of *Gnrh2*, *Fshβ*, *Lhβ*, *Fshr*, and *Lhr* genes in *Carassius auratus* with different ploidy and methylation of promoter regions, methylation levels of some promoters of the above genes were detected, and the results are shown in **Figure 5**. During the breeding season, methylation levels in the *Gnrh2*, *Fshβ*, and *Fshr* promoter regions were significantly reduced in 3nCC compared to 2nCC (*P*<0.05). After triploidization, the methylation levels of the *Gnrh2* promoter region were 0.489 and 0.6 in 3nCC females and males, respectively, and 0.822 and 0.789 in 2nCC females and males, respectively.

**Figure 6:**
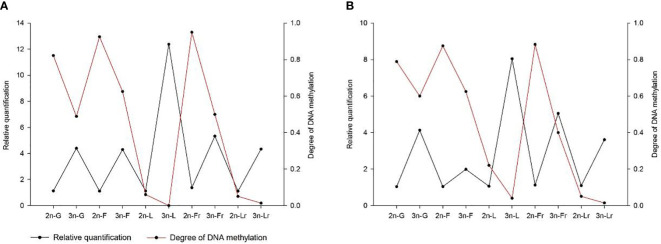
Correlation between the gene expression level and the methylation level of gene promoter. **(A)** 2nCC and 3nCC (female); **(B)** 2nCC and 3nCC (male); G stands for *Gnrh2* gene; F stands for *Fshβ* gene; L stands for *Lhβ* gene; Fr stands for *Fshr* gene; Lr stands for *Lhr* gene.

Methylation levels of *Fshβ* gene promoter region, the methylation levels of the promoter region of the *Fshr* gene were 0.5 and 0.4 in 3nCC females and males, respectively, and 0.95 and 0.883 in 2nCC females and males, respectively. After triploidization, the methylation levels of the promoter regions of the *Lhβ* and *Lhr* genes were decreased (but not significantly different) in 3nCC. The methylation levels of the *Lhβ* promoter region were 0 and 0.04 in 3nCC females and males, respectively, 0.06 and 0.22 in 2nCC females and males in the expression and localization analysis of HPG axis genes in *Carassius auratus* with different ploidy. Methylation levels in the *Lhr* promoter region were 0.013 and 0.014 in 3nCC females and males, respectively, and 0.05 and 0.05 in 2nCC females and males, respectively. A correlation analysis was performed between the HPG axis gene expression levels and the promoter methylation levels of the two ploidy *C. auratus* during the breeding season (**Figure 6**). The two were approximately negatively correlated (both females and males).

### 3.4 Sequence results of CpG-enriched regions of partial promoters of *Gnrh2*, *Gthβ* and *Gthr* genes

**Figure 7:**
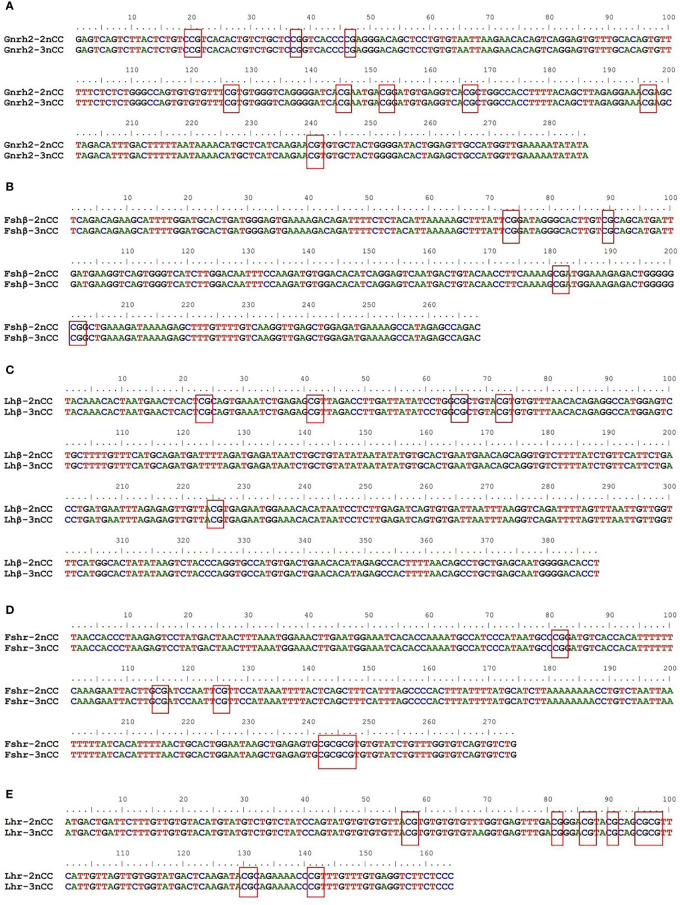
Sequence structure of CpG-rich region of gene promoter. The red boxes show the CpG sites in the promoter region of each gene. **(A)** Sequence comparison of 2nCC and 3ncc in *Gnrh2*. **(B)** Sequence comparison of 2nCC and 3ncc in *Fshβ*. **(C)** Sequence comparison of 2nCC and 3ncc in *Lhβ*. **(D)** Sequence comparison of 2nCC and 3ncc in *Fshr*. **(E)** Sequence comparison of 2nCC and 3ncc in *Lhr*.

BioEdit software was used to analyze the sequences of CpG-enriched regions of partial promoters of HPG axis-related genes in 2nCC and 3nCC. The results are shown in **Figure 7**. Among them, the number of CpG sites in the *Gnrh2* promoter region (**Figure 7A**), *Fshβ* (**Figure 7B**), *Lhβ* (**Figure 7C**), *Fshr* (**Figure 7D**), and *Lhr* (**Figure 7E**) genes were 9, 4, 5, 6, and 8, respectively.

### 3.5 Pituitary and gonadal structure of diploid and triploid *Carassius auratus*

**Figure 8:**
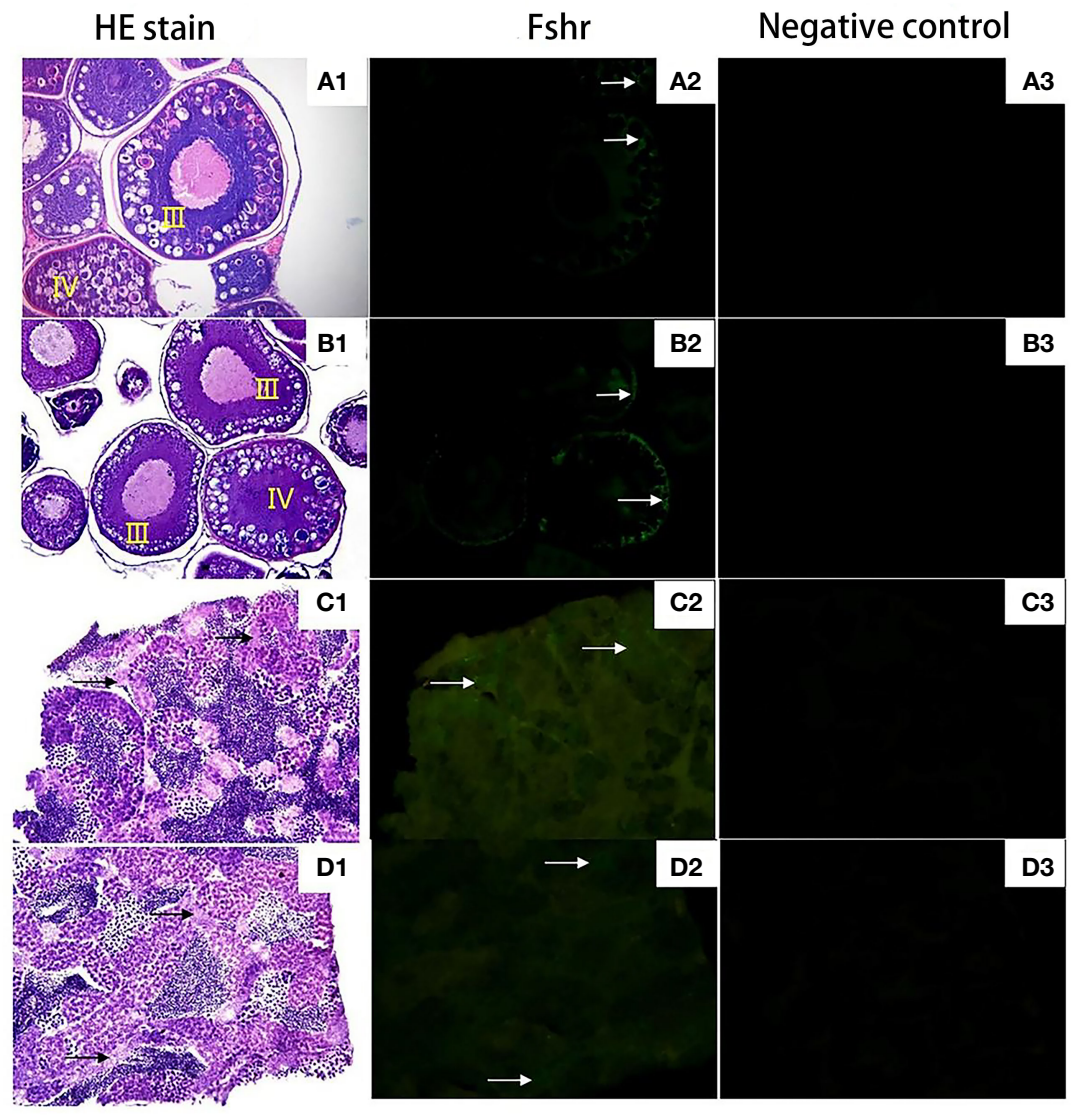
Expression localization of *Fshr* gene in gonads of 2nCC and 3nCC. **(A1)** 2nCC ovary, **(B1)** 3nCC ovary (20× magnification); **(C1)** 2nCC testis, **(D1)** 3nCC testis (40× magnification); **(A2)** Graph of fluorescence in situ hybridization results for the *Fshr* gene in 2ncc ovary, **(B2)** Graph of fluorescence in situ hybridization results for the *Fshr* gene in 3ncc ovary, **(D2)** Graph of fluorescence in situ hybridization results for the *Fshr* gene in 2ncc testis, **(E2)** Graph of fluorescence in situ hybridization results for the *Fshr* gene in 3ncc testis; **(A3)** 2nCC ovary fluorescence in situ hybridization negative control, **(B3)** 3nCC ovary fluorescence in situ hybridization negative control, **(C3)** 2nCC testis fluorescence in situ hybridization negative control, **(D3)** 3nCC testis fluorescence in situ hybridization negative control; white arrows indicate the localization of *Fshr* gene

**Figure 8** shows that both 2nCC and 3nCC have normal pituitary structures, and there are purple-red or blue-purple stained GTH cells in the pituitary region of both fish.

**Figures 8A1, B1** show that the ovary structure of the two fish developed as expected, and the development levels were essentially synchronized. Many phase III and IV oocytes were found in the ovaries, and some phase II oocytes were found in the ovaries. **Figures 8C1, D1** show that the testis structure of 2nCC and 3nCC also developed as expected, and several spermatocytes and sperm cells appeared in the seminiferous tubules.

### 3.6 Localization of *Lhβ* and *Fshβ* in pituitary tissue

**Figure 9:**
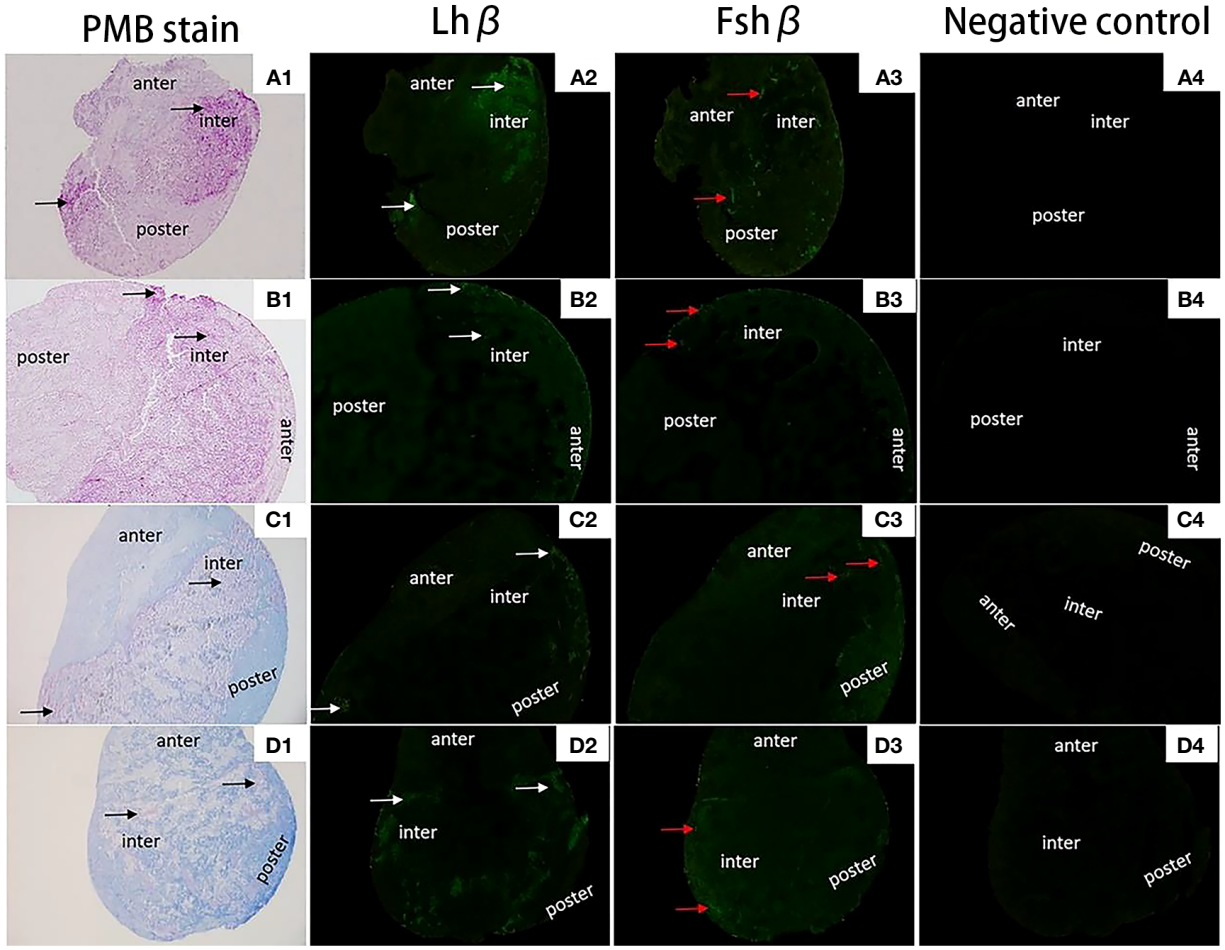
Expression localization of *Lhβ* and *Fshβ* genes in the pituitary of 2nCC and 3nCC. **(A1)** 2nCC (female pituitary); **(B1)** 3nCC (female pituitary); **(C1)** 2nCC (male pituitary); **(D1)** 3nCC (male pituitary) (10x magnification); **(A2)** Graph of fluorescence in situ hybridization results for the *Lhβ* gene in 2nCC female pituitary; **(B2)** Graph of fluorescence in situ hybridization results for the *Lhβ* gene in 3nCC female pituitary; **(C2)** Graph of fluorescence in situ hybridization results for the *Lhβ* gene in 2nCC male pituitary; **(D2)** Graph of fluorescence in situ hybridization results for the *Lhβ* gene in 3nCC male pituitary (10x magnification); **(A3)** Graph of fluorescence in situ hybridization results for the *Fshβ* gene in 2nCC female pituitary; **(B3)** Graph of fluorescence in situ hybridization results for the *Fshβ* gene in 3nCC female pituitary; **(C3)** Graph of fluorescence in situ hybridization results for the *Fshβ* gene in 2ncc male pituitary; **(D3)** Graph of fluorescence in situ hybridization results for the *Fshβ* gene in 3nCC male pituitary (10x magnification); **(A4)** 2nCC female pituitary fluorescence in situ hybridization negative control; **(B4)** 3nCC female pituitary fluorescence in situ hybridization negative control; **(C4)** 2nCC male pituitary fluorescence in situ hybridization negative control; **(D4)** 3nCC male pituitary fluorescence in situ hybridization negative control (10x magnification); black arrows indicate the gonadotropin (GTH) cells in the mid-hypophysis region stained purple; white arrows indicate the localization of *Lhβ* gene; red arrows indicate the localization of *Fshβ*gene.

The localization of *Fshβ* and *Lshβ* genes in the pituitary (**Figures 9 A1, B1, C1, D1**) tissues of diploid and triploid wild crucian carp was detected by immunofluorescence technique. In the GTH cells of the adenohypophysis of the diploid *C. auratus*, green fluorescence was observed in the area marked by the white arrow; a positive hybridization signal appeared (**Figures 9 A2, B2, C2, D2**). In contrast, no green fluorescence was observed in the negative control group (**Figures 9 A4, B4, C4, D4**); no positive hybridization signals appeared. The immunofluorescence section of the *Fshβ* gene showed that the expression site of the *Fshβ* gene was approximately the same as that of the *Lhβ* gene and located in GTH adenohypophysis cells of the two fish. It can be observed that the area indicated by the red arrow in the figure has green fluorescence, and a positive hybridization signal appears (**Figures 9A3, B3, C3, D3**). No green fluorescence was seen in the control group. There was no positive hybridization signal.

### 3.7 Localization of *Fshr* in ovarian and testes

Green fluorescence was observed in Sertoli cells of *C. auratus* diploid and follicular and granulosa cells of oocytes that developed in stages III and IV (**Figures 8A2, B2, C2, D2**, the area indicated by the white arrow), while no green fluorescence was found in the negative control group; no positive hybridization signals (**Figures 8A3, B3, C3, D3**).

## 4 Discussion

Polyploidy is widespread in fish evolution and is one of the main drivers of fish genome evolution, often accompanied by gene duplication and loss (22, 23). Genes can undergo an increase in copy number after polyploidy (24). Several studies analyzed the *Sox* gene family of natural and artificial polyploid fish and found that most *Sox* genes increased copy number after polyploidy events (25). After the number of gene copies increases, some may undergo pseudogenization, neofunctionalization, or subfunctionalization due to the pressures of natural evolution and selection (26). Gene loss can occur when one or more sets of genomes are added to a species to overcome the adverse effects of genomic changes. Some polyploids revert to a diploid state, such as 20% of the zebrafish genome. This gene remains replicated (27). In this study, the *Fshβ* and *Lhβ* genes had only one copy in 2nCC and 3nCC. That is, the number of gene copies did not increase after triploidization. It is speculated that the above two genes may have been lost during genome duplication.

Due to abnormal gonadal development or abnormal chromosomal behavior during meiosis, triploid fish are generally considered infertile. Gonadal development is dependent on the synthesis and secretion of sex hormones. Therefore, insufficient sex hormone secretion may reduce fertility or infertility in fish (21). Normal expression of HPG axis-related genes may regulate the formation of sex hormones, and a sufficient amount of sex hormones may ensure the normal progress of important life activities, such as fish gonadal development and gamete maturation. In this study, compared with 2nCC, the copy numbers of *Gnrh2*, *Fshr*, and *Lhr* genes were higher in 3nCC. Increasing the number of copies of these genes may promote gene expression so that 3nCC can produce enough sex steroid hormones. Thus, the fertility decline or infertility dilemma caused by abnormal synthesis and secretion of sex hormones in triploid fish is broken. Polyploidization can also cause changes in gene structure, which usually involve single-base mutations, insertions, or deletions of short and long sequences (28). For example, an analysis of different **Sox*9a* copies in RCC and 4nRR found that the four **Sox*9a* genes in 4nRR had single nucleotide site mutations, base sequence insertions, and base sequence insertion/deletion phenomena (29). Herein, base site mutations in *Gnrh2*, *Fshr*, and *Lhr* were observed in 3nCC, and deletion was present in the *Lhr* gene, indicating that triploidization caused no structural changes in the gene. Site mutations in *Gnrh2*, *Fshr*, and *Lhr* genes were observed in 3nCC, but most are unknown mutations. The nucleotide sequence similarity of the above genes is more than 93.8%. The biologically active decapeptide and proteolysis site of the *Gnrh2* gene, the cysteine-residue site in *Fshβ* and *Lhβ* genes, and the glycosylation site “NIS” are highly conserved, and the high conservation of gene sequences suggests their functional importance. After triploidization, no significant changes were observed in the structure of HPG axis genes, which greatly ensured the stable expression of the above genes in the corresponding tissues. This may be one of the potential reasons for the fertility recovery of 3nCC. During the formation of polyploidy, genomic fusion may generate many redundant genes. Therefore, the reproduction mode, gene structure (30), and gene expression (31) change in polyploidy.

In general, polyploidy may positively regulate gene expression due to higher gene copy numbers. That is, an increase in ploidy increases gene expression. Studies on maize with different ploidy found that the expression of most genes was positively correlated with ploidy. For example, sucrose synthase expression in maize was proportional to the increase in gene copy number (32). Studies of biological enzyme activity have shown a positive correlation between enzyme activity and an increase in gene copy number (33). In fish, the expression of HPG axis-related genes was significantly higher in autotetraploid *C.auratus* than in parent diploid *C. auratus red var.* (22). Herein, the expression of HPG axis-related genes was significantly higher in 3nCC than in 2nCC (*P*<0.05). Therefore, it is speculated that the upregulation of genes related to the HPG axis following triploidization may be directly associated with higher gene copies in 3nCC. Studies have shown that compared with diploid and tetraploid, the expression of *Fshr* and *Lhr* genes in triploid allotriploid *C.auratus* is relatively reduced. The synthesis and secretion of sex steroid hormones are also reduced, which ultimately affects the development of triploid gonads and gametes. Mature triploid allotriploid *C.auratus* showed sterile reproductive characteristics (34). This study observed a significant increase in the expression of the HPG axis gene after triploidization, which resulted in 3nCC producing sufficient gonadotropins. This, in turn, was affected by the expression and localization of HPG axis-related genes in *C. auratus* with varying ploidy. The gonadotropin ligands (*Fshβ* and *Lhβ*) combine with their respective analytes (*FSHR* and *LHR*), allowing 3nCC to synthesize and secrete sufficient sex steroid hormones. This facilitates the growth and reproduction of individuals and promotes the development of gonads and the maturation of gametes in 3nCC.

Many factors affect the expression of polyploid genes. In addition to gene copy numbers, epigenetic regulation and mutual regulation between genes exist. Among them, the regulation of gene expression by methylation in gene promoter regions has received extensive attention. Methylation modification may affect DNA conformation, chromatin structure, or gene transcription (35), inhibiting or silencing gene expression. By detecting gene expression and DNA transposon methylation in parent diploid indica and autotetraploid rice, no significant difference was found between the expression of most genes in hyperploid rice and parents. This suggests that the high DNA methylation of transposons in high-ploidy rice affects the expression of their associated genes (36). In this study, the RT-qPCR and BS-PCR results of HPG axis-related genes in *C. auratus* with different ploidy revealed a negative correlation between the two variables (were consistent in female and male individuals), consistent with previous findings on the relationship between Ph1 and Dmc1 expression and promoter methylation in autotriploid *C.auratus (* 37). The results showed that after triploidization, the methylation of the gene promoter could have a certain regulatory effect on gene expression. The increased expression of HPG axis-related genes and decreased methylation levels in the promoter region may be combined to promote the production and secretion of sex steroid hormones, subsequently regulating the gonadal development of 3nCC. The hypothalamus-pituitary-gonadal axis regulates the gonadal development and gamete maturation of fish (38), with the pituitary and ovary playing a crucial role as endocrine organs. They directly participate in important life activities, such as fish growth, development, and reproduction. The structural integrity of pituitary and gonadal tissue and the normal functioning of its functions are the basis for the normal reproduction of fish. In this study, pituitary tissue sections of 2nCC and 3nCC were stained with periodic acid-Schiff reagent-methyl blue staining, and the results showed that GTH cells that stained purple-red were located in the middle pituitary region. The results of this experiment are consistent with previous findings, indicating that the distribution of GTH in the pituitary tissue of *C. auratus red var.* and autotetraploid *C.auratus* is similar (22). The maintenance of a normal pituitary structure and stable distribution of gonadotropin-releasing hormone (GTH) cells in 3nCC is crucial for the proper functioning of the pituitary gland. This, in turn, facilitates normal endocrine activities and effectively promotes the growth, development, and maturation of germ cells within 3nCC. Fish fertility is not only related to the pituitary structure but also to the gonad structure and development level.

Observing the development of fish testes and ovaries during the breeding season can determine whether they have normal reproductive capacity. Gonad tissue sections of *C.auratus* with different ploidy during the breeding season (April) were examined. It was observed that the testis and ovary structures of 2nCC and 3nCC were normal. The normal fertility of 3nCC provides a basis for further study. Some studies have reported that *Fshβ* and *Lhβ* genes are only expressed in the pituitary tissue of autotetraploid *C.auratus*, while *Fshr* and *Lhr* genes are only expressed in the gonadal tissue of fish (39). Normal expression of *Fshβ* and *Lhβ* genes can produce gonadotropin *Gthβ* (*Fshβ* and *Lhβ*), and *Gthβ* can combine with gonadotropin *Gthr.* (*FSHR* and *LHR*) to promote biological gonadal development and ovulation (39, 40) investigated the expression and localization of *Gthβ* (*Fshβ* and *Lhβ*) and *Gthr* (*Fshr* and *Lhr*) genes in *C.auratus* with different ploidy using *in situ* hybridization. The results showed that both *Fshβ* and *Lhβ* were specifically expressed in fish. In pituitary tissue, *Fshr* and *Lhr* genes are mainly expressed in fish ovarian follicles, granulosa cells, and outside the radial membrane of oocytes (39), and in male testis, they are expressed in stromal cells and Sertoli cells. Follicular and granulosa cells are usually involved in fish estrogen production, while Sertoli cells are involved in sperm formation and maturation and synthesis and secretion of androgen-binding proteins. Immunofluorescence results showed that triploidization did not change the localization of *Fshβ*, *Lhβ*, and *Fshr* genes in the corresponding 2nCC and 3nCC tissues. The expression of *Fshr* in ovarian follicular cells may contribute to estrogen production and secretion, whereas its expression in testis-supporting cells may contribute to sperm development and maturation. The stability of these gene localizations greatly ensures their normal functioning. These research findings provide important cellular biological evidence for the normal process of gonadal development and gamete maturation in 3nCC.

In summary, the characteristics of *Gnrh2, Gthβ* and *Gthr* gene sequence, expression level, promoter methylation level, and expression localization in 2nCC and 3nCC pituitary and gonad tissue were analyzed.. In this study, differences in gene localization were analyzed, which revealed that no changes in coding sequences of *Fshβ* and *Lhβ* genes occurred occur after triploidization. The coding sequence of a gene is the part that contains the instructions for making a protein. If these sequences remain unchanged, it suggests that the proteins encoded by these genes would be the same in 2nCC and 3nCC organisms, at least in terms of their amino acid sequences. In the study of the *Sox* gene family in autotetraploid crucian carp by Huang Xu and others, it was also found that some genes within the *Sox* family (*Sox*6, *Sox*11, *Sox*17, and *Sox*32) retained their original sequences after polyploidization and did not duplicate (25). This indicates that not all genes increase in number after an increase in chromosome count. In contrast, the number of copies of the coding regions of *Gnrh2*, *Fshr*, and *Lhr* genes increased, leading to significant differentiation in structure and a significant increase in transcription level. In addition, the methylation level of the promoter region decreased, and the location of *Gthβ* (*Fshβ*, *Lhβ*) and *Fshr* genes remained unchanged. A decrease in methylation levels is usually associated with an upregulation of gene expression. In gene regulation, DNA methylation is an epigenetic mechanism that modulates gene expression by adding methyl groups to specific sites on the DNA molecule. In many cases, methylation of the promoter region of a gene leads to gene silencing or a reduction in expression levels, as methylation can prevent the binding of regulatory proteins such as transcription factors. Therefore, when the methylation level of a gene’s promoter region is reduced, it typically indicates that transcriptional barriers have been removed, making the gene more accessible to the transcriptional machinery and thereby resulting in increased levels of gene expression (41). Those changes are critical in many biological processes, such as regulating organisms (42), development (21) and response to environmental changes (43). These findings provide both cellular and molecular biology evidence for normal reproductive activities, such as gonad development and gamete maturation, in triploid *Carassius auratus* and offer theoretical support for understanding the fertility recovery of triploid *Carassius auratus*. They also have important implications for the protection of quality resources and the breeding of fish polyploidy.

## Data availability statement

The datasets presented in this study can be found in online repositories. The names of the repository/repositories and accession number(s) can be found below: https://www.ncbi.nlm.nih.gov/, OQ849750, https://www.ncbi.nlm.nih.gov/, OR039290, https://www.ncbi.nlm.nih.gov/, OR039291, https://www.ncbi.nlm.nih.gov/, OR039292, https://www.ncbi.nlm.nih.gov/, OR039293, https://www.ncbi.nlm.nih.gov/, OR039294, https://www.ncbi.nlm.nih.gov/, OR039295, https://www.ncbi.nlm.nih.gov/, OR039296, https://www.ncbi.nlm.nih.gov/, OR039297, https://www.ncbi.nlm.nih.gov/, OR039298, https://www.ncbi.nlm.nih.gov/, OR039299, https://www.ncbi.nlm.nih.gov/, OR039300, https://www.ncbi.nlm.nih.gov/, OR039301, https://www.ncbi.nlm.nih.gov/, OR039302, https://www.ncbi.nlm.nih.gov/, OR039303.

## Ethics statement

Fish treatments were carried out according to the recommendations in the Guidelines for the Care and Use of Laboratory Animals of the National Advisory Committee for Laboratory Animal Research in China and approved by the Animal Care Committee of Hunan Normal University (Permit Number: 4237). Individual 2nCC and 3nCC were randomly selected. All samples were cultured in open pools (0.067 ha) with suitable pH (7.0-8.5), water temperature (22-24°C), dissolved oxygen content (5.0-8.0 mg/L) and adequate forage at the State Key Laboratory of Developmental Biology of Freshwater Fish, Hunan Normal University, China. All fish were anesthetized with 100 mg/L MS-222 (Sigma-Aldrich, St Louis, MO, USA) before blood collection. The study was conducted in accordance with the local legislation and institutional requirements.

## Author contributions

XWX: Methodology, Writing – original draft, Writing – review & editing. LY: Methodology, Formal analysis. XD: Project administration, Validation. QX: Project administration, Validation. XH: Project administration, Validation. CW: Project administration, Validation. YZ: Project administration, Validation. XL: Project administration, Validation. YXZ: Project administration, Validation. XDX: Project administration, Validation. QQ: Conceptualization, Writing – review & editing. SL: Conceptualization, Writing – review & editing.
